# Specialties

**Published:** 1869-12

**Authors:** 


Specialties.—A distinguished correspondent of this month writes us as follows:

Old things are fast passing away, and he who would stand still and cling to antiquated ideas must needs be soon lost in this age of telegraphs and railroads. We are prompted to make these remarks by the rapid and long strides which the science of medicine is daily making. In the days of our grandfathers, when an individual put up his sign as a Doctor, he professed and in truth advertised himself to pull teeth, doctor eyes, cut off legs, etc.; things have somewhat changed since then. The extent of the field of learning occupied by medicine is so great, that it is impossible for one mind in the three score years and ten, to master all of the many difficult problems on the chess board of life. Hence, the natural tendency to division of labor, from whence arise the specialties in medicine. Since then, this necessity exists, why should the great law-making power of the Doctors condemn this division of labor? It does so by putting its seal of outlawry upon all those who dare, by either sign or any other modest way tell the public what they are. We know that it requires years of intense study to make oneself proficient in any particular class of diseases, especially those of the eye and ear. Ought you then to expect one who has spent the best years of his life in such a course, " to hide his light under a bushel?" yet such is the effect of the code of ethics, for he cannot, make himself known in the light of his specialty, for fear of being tabooed by the high dignitaries of the American Medical Association. A change is demanded, not only in justice to those whose inclination may have led them in some special channel, but also in justice to the great public whom it is the special duty of the medical profession to protect. By pursuing the present course, think of the untold injuries which may be, and are daily inflicted by these advertising pretenders who see only the almighty dollar in their every act. We are not only for permitting the man of science who professes a specialty, to announce himself as such, but to compel him to do it—or else the general practitioner will be destroyed. Let the specialist be unmasked, and sell for whatever he will bring in the market. He has preyed upon the vitals of the general practitioner long enough. Strip him of the garb of greatness, with which mystery has invested him, and force him to appear in his true character. Force him to come out and fight the great enemy under his little brigade colors alone. Don't let him wander all over the field and take a stray shot here and another there, wherever he can find a good place fitted up by an old-fashioned general practitioner. Let the surgeon announce himself as such, the aurist and oculist in like manner, but hold them to their bargain, and do not let them trespass upon the field of the general practitioner. By pursuing any other course, you put the latter class completely at the mercy of the specialist. To illustrate:—Let us suppose a case of serious injury occurring; the family physician is sent for—he calls to his aid a surgeon, who, as a matter of course, leads in the case, thus throwing the family physician completely in the back-ground. All the neighbors witness this ignoring of their great man, and that too, by his consent. What is the result? His sceptre departs from him, and it matters not what occurs in that region thereafter, a belly-ache or what not, the great surgeon is sent for. How often does it happen that good men are thus ridden over rough-shod by those who are their inferiors, through the influence of the glitter of an amputating knife. Now suppose you force this surgeon to announce himself as such, the people are educated by this fact, and expect, in all cases of injury, that his assistance will be demanded. He receives the credit to which he is entitled, and no one loses, for he is fenced in by his specialty.

Therefore, we say, not only permit them, but compel them to come forth from the hiding place in which they are now cooped by Article——of the Code of Ethics.—*Nashville Journal of Medicine and Surgery.*

